# Efficacy of Patient Activation Interventions With or Without Financial Incentives to Promote Prescribing of Thiazides and Hypertension Control: A Randomized Clinical Trial

**DOI:** 10.1001/jamanetworkopen.2018.5017

**Published:** 2018-12-14

**Authors:** Peter J. Kaboli, M. Bryant Howren, Areef Ishani, Barry Carter, Alan J. Christensen, Mark W. Vander Weg

**Affiliations:** 1 Center for Comprehensive Access & Delivery Research & Evaluation, Veterans Affairs Iowa City Healthcare System, Iowa City, Iowa; 2 Department of Internal Medicine, The University of Iowa Carver College of Medicine, Iowa City; 3 Department of Psychological and Brain Sciences, The University of Iowa College of Liberal Arts and Sciences, Iowa City; 4 Center for Epidemiology and Clinical Research, Minneapolis Veterans Affairs Medical Center, Minneapolis, Minnesota; 5 Section of Nephrology, Department of Medicine, Minneapolis Veterans Affairs Medical Center, Minneapolis, Minnesota; 6 Division of Renal Diseases and Hypertension, University of Minnesota, Minneapolis; 7 Department of Pharmacy Practice and Science, The University of Iowa College of Pharmacy, Iowa City; 8 Department of Family Medicine, The University of Iowa Carver College of Medicine, Iowa City

## Abstract

**Importance:**

Evidence-based guidelines recommend thiazide diuretics as a first-line therapy for uncomplicated hypertension; however, thiazides are underused, and hypertension remains inadequately managed.

**Objective:**

To test the efficacy of a patient activation intervention with financial incentives to promote thiazide prescribing.

**Design, Setting, and Participants:**

The Veterans Affairs Project to Implement Diuretics, a randomized clinical trial, was conducted at 13 Veterans Affairs primary care clinics from August 1, 2006, to July 31, 2008, with 12 months of follow-up. A total of 61 019 patients were screened to identify 2853 eligible patients who were not taking a thiazide and not at their blood pressure (BP) goal; 598 consented to participate. Statistical analysis was conducted from December 1, 2017, to September 12, 2018.

**Interventions:**

Patients were randomized to a control group (n = 196) or 1 of 3 intervention groups designed to activate patients to talk with their primary care clinicians about thiazides and hypertension: group A (n = 143) received an activation letter, group B (n = 128) received a letter plus a financial incentive, and group C (n = 131) received a letter, financial incentive, and a telephone call encouraging patients to speak with their primary care clinicians.

**Main Outcomes and Measures:**

Primary outcomes were thiazide prescribing and BP control. A secondary process measure was discussion between patient and primary care clinician about thiazides.

**Results:**

Among 598 participants (588 men and 10 women), the mean (SD) age for the combined intervention groups (n = 402) was 62.9 (8.8) years, and the mean baseline BP was 148.1/83.8 mm Hg; the mean (SD) age for the control group (n = 196) was 64.1 (9.2) years, and the mean baseline BP was 151.0/83.4 mm Hg. At index visits, the unadjusted rate of thiazide prescribing was 9.7% for the control group (19 of 196) and 24.5% (35 of 143) for group A, 25.8% (33 of 128) for group B, and 32.8% (43 of 131) for group C (*P* < .001). Adjusted analyses demonstrated an intervention effect on thiazide prescribing at the index visit and 6-month visit, which diminished at the 12-month visit. For BP control, there was a significant intervention effect at the 12-month follow-up for group C (adjusted odds ratio, 1.73; 95% CI, 1.06-2.83; *P* = .04). Intervention groups exhibited improved thiazide discussion rates in a dose-response fashion: group A, 44.1% (63 of 143); group B, 56.3% (72 of 128); and group C, 68.7% (90 of 131) (*P* = .004).

**Conclusions and Relevance:**

This patient activation intervention about thiazides for hypertension resulted in two-thirds of patients having discussions and nearly one-third initiating a prescription of thiazide. Adding a financial incentive and telephone call to the letter resulted in incremental improvements in both outcomes. By 12 months, improved BP control was also evident. This low-cost, low-intensity intervention resulted in high rates of discussions between patients and clinicians and subsequent thiazide treatment and may be used to promote evidence-based guidelines and overcome clinical inertia.

**Trial Registration:**

ClinicalTrials.gov Identifier: NCT00265538

## Key Points

**Question:**

What is the effect of a patient activation intervention using patient education, financial incentives, and reminder telephone calls to improve care for hypertension?

**Findings:**

In this randomized clinical trial of 598 patients, those who received the intervention were more likely to discuss hypertension treatment with their primary care clinicians and receive evidence-based care. In a dose-response manner, adding financial incentives and reminder telephone calls had incremental effects, and significantly improved rates of attainment of goal blood pressure were achieved after 12 months.

**Meaning:**

To attain high levels of blood pressure control and guideline-concordant therapy, engaging patients through patient activation interventions appears to be an effective, low-cost intervention.

## Teaser

This randomized clinical trial tests the efficacy of patient activation interventions with or without financial incentives to promote prescribing of thiazide diuretics in patients with hypertension and improve the quality of hypertension management.

## Introduction

Hypertension is the most common treatable cardiovascular risk factor in the United States, affecting almost 1 in 3 individuals.^1^ Despite improvements in the detection and management of hypertension, its prevalence persists, and the percentage of patients whose hypertension is controlled remains unacceptably low.^2,3,4,5,6,7,8,9,10^ National estimates suggest that at least half of patients with hypertension have uncontrolled blood pressure (BP); studies by the Department of Veterans Affairs have shown similarly poor control of BP.^6,9,10,11^ New guidelines published in 2017 suggest a goal BP of less than 130/80 mm Hg for most patients.^12^ If these lower target guidelines are adopted by health systems, BP will be uncontrolled in far higher numbers of patients with hypertension.

In addition to suboptimal BP control, rates of guideline-concordant therapy also remain low despite evidence-based guidelines from the Joint National Committee (JNC) on Prevention, Detection, Evaluation, and Treatment of High Blood Pressure (formerly JNC 8, renamed the 2014 Guidelines).^13,14^ Based on these and other guidelines as well as the large clinical trial Anti-Hypertensive and Lipid-Lowering Therapy to Prevent Heart Attack Trial (ALLHAT),^15^ thiazide diuretics are recommended as a first-line therapy for uncomplicated hypertension and frequently should be added to treatment regimens to improve BP control. At the time of this study, the Seventh Report of the JNC on Prevention, Detection, Evaluation, and Treatment of High Blood Pressure^16^ comprised the prevailing guidelines and advocated thiazide diuretics as first-line therapy for uncomplicated hypertension, whereas the 2014 Guidelines recommend selection of treatment from among 4 classes, including thiazides.^14^

Numerous strategies have been studied and promoted to improve treatment of hypertension, including primary care clinician–based interventions, such as academic detailing and promotion of guideline adherence, as well as patient self-management; web-based interventions that use secure communication between patients and clinicians; and systems-based interventions, such as computerized reminders or institution of interdisciplinary care teams.^17,18,19,20,21,22^ Meta-analyses of quality improvement strategies to improve care have found that targeting patients or adding a health care team member, such as a nurse or pharmacist, resulted in the largest reductions in BP.^23,24,25,26^ However, many interventions studied have had disappointing results or may be impractical for implementation in everyday clinical practice.^18,19,21,23^ Interventions providing general educational information on hypertension have generally proven to be inadequate.^27,28^

Achieving more aggressive BP goals will require complex and innovative interventions. One promising facet of the quality gap paradigm is the role of the patient to promote evidence-based health care. Direct-to-consumer marketing has been used by the pharmaceutical industry to motivate discussions between patients and physicians to affect prescribing.^29,30,31^ Fundamentally, direct-to-consumer marketing seeks to increase patient activation—a term referring to the process of imparting knowledge, skills, and confidence such that patients become more active and informed participants in the delivery of health care.^32^ Although patient activation is not new, few patient activation interventions have been developed,^33^ which has been highlighted as health care entities seek to increase quality while decreasing costs.^34^ Patient activation interventions have shown promise on both outcomes and cost,^33,35^ and the field of behavioral economics is increasingly being used to “nudge” care improvements.^36^

The goal of this study was to test the efficacy of a low-cost, low-intensity patient activation intervention to increase use of thiazide diuretics and improve the quality of hypertension management. Our strategy included a 3-tiered intervention including customized patient education and the addition of financial incentives and health educator telephone calls designed to encourage patient engagement at the clinic encounter. It was hypothesized that thiazide use would be higher within each intervention arm and hypertension control would improve at 6 and 12 months compared with usual care.

## Methods

### Participants

Study participants were sampled from all patients who received primary care at 13 Veterans Affairs outpatient clinics affiliated with the Iowa City and Minneapolis Veterans Affairs Medical Centers (N = 61 019). Eligible participants included patients with hypertension who were not taking a thiazide and were not at an appropriate BP goal at the 2 most recent clinic visits (systolic BP ≥140 mm Hg [130 mm Hg for those with diabetes or renal insufficiency] or diastolic BP ≥90 mm Hg [80 mm Hg for those with diabetes or renal insufficiency]) (see Box for additional criteria). Automated protocols were applied to the electronic medical record to identify eligible participants. The Veterans Affairs Project to Implement Diuretics (VAPID) Study was approved by the institutional review boards at the Iowa City and Minneapolis Veterans Affairs Medical Centers. Patients provided written informed consent. Race/ethnicity was determined based on patient self-report and recorded in the electronic medical record. This study followed the Consolidated Standards of Reporting Trials (CONSORT) reporting guidelines.

Box. **Inclusion and Exclusion Criteria**

**Inclusions for EMR Screening (All Patients With a Scheduled Primary Care Appointment in the Following 3 Months)**

- Hypertension diagnosis (*ICD-9* codes 401.XX-405.XX)
- Age <80 years
- ≥2 Veterans Affairs clinic visits in past 12 months

**Exclusions From EMR Screening (in Prior 12 Months)**

**Active Medications**

- Thiazide diuretics (ie, hydrochlorothiazide, chlorthalidone, chlorthiazide, indapamide, or metolazone), loop diuretic (ie, furosemide, torsemide, or budesonide), lithium, and allopurinol

**Diagnoses**

- Gout (*ICD-9* codes 274.00-274.99)

**Laboratory Values**

- Hypokalemia (serum potassium, <3.5 mEq/L)
- No serum creatinine or calculated estimated glomerular filtration rate <30 mL/min

**Other**

- Resident of a long-term care facility
- No telephone
- Thiazide or sulfa allergy or prior adverse drug event

**Blood Pressure (Either Diastolic or Systolic)**

- Type 1 or type 2 diabetes and blood pressure <130/80 mm Hg on 2 most recent visits
- Renal insufficiency (creatinine clearance, 30-59 mL/min) and blood pressure <130/80 mm Hg on 2 most recent visits
- No diabetes and blood pressure <140/90 mm Hg

**Exclusions From EMR Screening**

- Congestive heart failure with ejection fraction <35%
- Life expectancy <6 months

**Exclusions After Patient Agrees to Participate (Telephone Interview)**

- Cognitive impairment (>2 errors on Short Portable Mental Status Questionnaire)

Abbreviations: EMR, electronic medical record; *ICD-9*, *International Classification of Diseases, Ninth Revision*.

SI conversion factor: To convert potassium to millimoles per liter, multiply by 1.0.

### Procedure

The study protocol is available in Supplement 1. Participants were recruited from August 1, 2006, to July 31, 2008, following a staged protocol that randomized primary care clinicians (ie, physicians, physician assistants, and nurse practitioners) to intervention and control groups and then selected eligible patients within these groups. Intervention and control group assignments were then made from sequentially numbered, sealed envelopes prepared in blocks of 20. On primary care clinician randomization, patients with a primary care appointment within 30 days were identified and screened using the electronic medical record. Eligible patients (n = 2853) were mailed a letter describing the study objectives, enrollment process, and data collection. One week later, patients were contacted by telephone to obtain informed consent and were administered a mental status questionnaire to exclude patients with cognitive impairment.^37^ Patients who consented were randomized (n = 598) to the control group or an intervention group. Enrollment ended when the target number of participants was enrolled. Complete follow-up was obtained by 572 participants (95.7%) at 6 months and 563 participants (94.1%) at 12 months (Figure).^38^ Sample sizes were selected to provide sufficient (≥80%) power to detect an 18% absolute difference in thiazide use between the control group and a single treatment group and a 15% difference between any 2 intervention groups if the within-clinician correlation is as high as 0.10, a conservative estimate.

**Figure.**
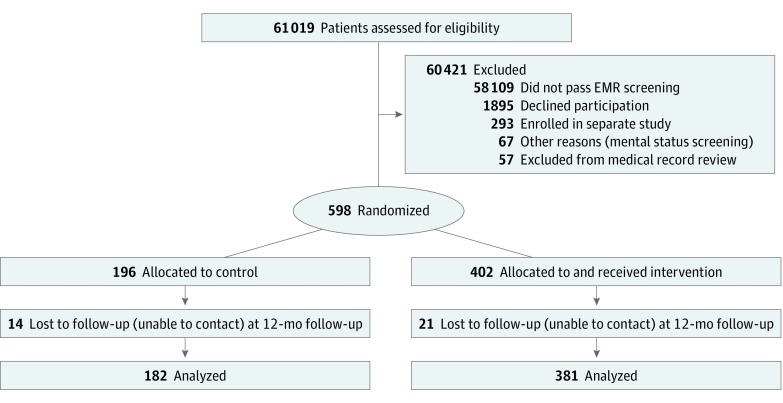
CONSORT 2010 Flow Diagram EMR indicates electronic medical record; and mental status screening indicates that the individual had more than 2 errors on the Short Portable Mental Status Questionnaire.

Patients randomized to the intervention were assigned to 1 of 3 intervention arms. Group A received a customized letter providing a Framingham Heart Study cardiovascular risk assessment^39,40^ mailed 1 to 3 weeks before their scheduled clinic appointment (hereafter referred to as the index visit). The sample letter is included in eAppendix 1 in Supplement 2. Information was customized for the patient’s treatment regimen, BP, and cardiovascular risk factors. The letter included prior BP readings, identified the patient’s BP goal, quantified the relative cardiovascular risk reduction that would occur with achieving the goal, and provided suggestions for reaching that goal, namely, addition of a thiazide. The letter requested that patients initiate a discussion with their primary care clinician about a thiazide and included a postcard for patients to have their clinicians sign and mail back, documenting whether they discussed thiazides during the visit.

Group B received the same patient education letter as well as a financial incentive to initiate a discussion with their primary care clinician. The incentive was a $20 payment that patients would be mailed on receipt of their signed postcard. For patients with a prescription copayment (70.4% of patients [421 of 598]), the letter also told them that their thiazide copayments for 6 months ($48) would be reimbursed on receipt of the postcard and confirmation that a thiazide was prescribed.

Group C received the same letter and financial incentives as group B plus a telephone call by a trained health educator 1 week before their clinic visit. The health educator discussed the mailed information, the patient’s role in initiating a discussion with the primary care clinician about BP medications, and potential strategies for initiating such conversations. A sample script is included in eAppendix 2 in Supplement 2.

### Outcomes

#### Thiazide Prescription

Initiation of a thiazide was determined by reviewing each patient’s progress note and medication list after the index visit. During the 6-month and 12-month follow-up telephone calls, medication lists were reviewed and thiazide use was confirmed. Previous research suggests that neither the electronic medical record nor patient interview alone has adequate sensitivity or specificity for determining medication use^41^; thus, both methods were used.

#### Blood Pressure

Baseline BP was determined from 2 clinic readings recorded in the electronic medical record at the index visit after randomization. If the BP was repeated, the lowest value was recorded. For the 6-month and 12-month follow-up visits, the reading from the closest clinic visit to those dates was recorded.

#### Patient-Initiated Discussion About Thiazides

Patient-initiated discussion about thiazides was a prespecified secondary end point. Two indices were used to determine if patients initiated a discussion with their primary care clinician regarding thiazide use. First, we examined the returned postcards. Because it was possible that patients had the discussion but did not return a postcard, we also examined clinic progress notes. A research assistant reviewed notes of patients who did not return a postcard and recorded whether there was evidence of a patient-initiated discussion of thiazides. Examples include, “Patient asked if he needs to start a low-dose water pill” and “He presents with a letter from the study group suggesting that a diuretic be started.” These 2 indices were then combined to determine if a discussion about thiazides occurred.

### Statistical Analysis

Statistical analysis was conducted from December 1, 2017, to September 12, 2018. The intervention and control groups were compared at baseline in terms of sociodemographic characteristics and other clinical variables of importance (eg, baseline BP, smoking status, mental status, and diabetic status) using analysis of variance, Kruskal-Wallis, or χ^2^ tests as appropriate. Variables showing a (conservative) significant difference between groups at *P* < .10 were included as covariates in adjusted analyses.

Because of the clustered nature of these data, statistical analyses must account for correlation for the observed results to be valid given that traditional regression models assume independence of observations.^42^ As such, analyses for the outcomes of thiazide prescription and attainment of goal BP used hierarchical logistic regression using the generalized linear mixed model procedure in SPSS, version 24.0 (SPSS Inc). Models incorporated random physician effects to account for within-physician clustering; clinic sites were included as fixed effects. Analyses tested separately after the index visit and 6- and 12-month follow-up visits if there was a significant difference between the log odds of thiazide prescription and attainment of goal BP, respectively, for the control group vs the mean of the 3 intervention groups. All *P* values were from 2-sided tests, and results were deemed statistically significant at *P* < .05. Significant differences were followed by pairwise comparisons (corrected using the Bonferroni method) to evaluate potential group differences between each level of the intervention and control on rate of patient-initiated thiazide discussion.

## Results

Throughout recruitment, 61 019 potential participants were screened and 2853 eligible participants (4.7%) were identified. Of these, 598 participants (588 men and 10 women) were randomized to either intervention (n = 402; group A, 143; group B, 128; and group C, 131) or control (n = 196) groups. The mean (SD) age for the combined intervention groups was 62.9 (8.8) years, and the mean BP at baseline was 148.1/83.8 mm Hg (Table 1). The mean (SD) age for the control group was 64.1 (9.2) years, and the mean BP at baseline was 151.0/83.4 mm Hg. Only race/ethnicity varied significantly between groups and was added as a covariate in all models; baseline BP was also included in regression models determining attainment of goal BP. No differential attrition by group was observed.

**Table 1.** Baseline Characteristics

Characteristic	Combined Intervention Groups (n = 402)	Control Group (n = 196)	*P* Value
Age, mean (SD), y	62.9 (8.8)	64.1 (9.2)	.51
Male, No. (% )	395 (98.3)	193 (98.5)	.58
Educational level, No. (%)			
Grade school	198 (49.3)	98 (50.0)	.61
High school	178 (44.3)	85 (43.4)	
Beyond high school	26 (6.5)	13 (6.6)	
Race/ethnicity, No. (%)			
White	260 (64.7)	137 (69.9)	.01
Other or unknown	137 (34.1)	54 (27.6)	
African American	5 (1.2)	5 (2.6)	
Blood pressure, mean (SD), mm Hg			
Systolic	148.1 (13.1)	151.0 (14.0)	.48
Diastolic	83.8 (10.0)	83.4 (10.6)	.42
Smoking status yes, No. (%)	107 (26.6)	57 (29.1)	.38
Diabetic status yes, No. (%)	100 (24.9)	44 (22.4)	.43
High adherence per Morisky Medication Adherence Scale–4 score, No. (%)	159 (39.6)	79 (40.3)	.95

The first primary outcome of interest was rate of thiazide prescription. The unadjusted thiazide prescription rate at the index visit was 9.7% for the control group (19 of 196) and 24.5% (35 of 143) for group A, 25.8% (33 of 128) for group B, and 32.8% (43 of 131) for group C (*P* < .001). At 6 months, the thiazide prescription rate increased to 16.7% (31 of 186) for the control group and 22.5% (31 of 138) for group A, 22.8% (28 of 123) for group B, and 29.6% (37 of 125) for group C (*P* = .04). At 12 months, thiazide prescription rates increased to 22.5% (41 of 182) for the control group, 25.7% (35 of 136) for group A, 27.0% (33 of 122) for group B, and 30.9% (38 of 123) for group C, but the differences were no longer significant (*P* = .43) (Table 2). Adjusted odds ratios for the 3 intervention groups relative to control are presented in Table 3 by time point, indicating that thiazide prescribing increased in a dose-response fashion, like thiazide discussion, at the index visit. No significant clustering owing to physicians or clinics was observed; that is, the intercluster correlation coefficient values were zero. The largest effect was seen in group C, with an adjusted odds ratio of 4.85 (95% CI, 2.60-9.05) compared with control. The effect size was reduced to 2.50 (95% CI, 1.42-4.41) at 6 months and 1.58 (95% CI, 0.93-2.67) at 12 months. Thiazide prescribing in the control group increased 2.5-fold (9.7% [19 of 196] to 22.5% [41 of 182]) during 12 months (Table 2).

**Table 2.** Unadjusted Results of Thiazide Prescribing and Blood Pressure Control at Index Visit, 6-Month Follow-up, and 12-Month Follow-up

Outcome and Group^a^	Index Visit (n = 598)		Follow-up			
			6 mo (n = 572)		12 mo (n = 563)	
	No./Total No. (%)	*P* Value^b^	No./Total No. (%)	*P* Value^b^	No./Total No. (%)	*P* Value^b^
Thiazide prescribing						
Control	19/196 (9.7)	<.001	31/186 (16.7)	.04	41/182 (22.5)	.43
Intervention A	35/143 (24.5)		31/138 (22.5)		35/136 (25.7)	
Intervention B	33/128 (25.8)		28/123 (22.8)		33/122 (27.0)	
Intervention C	43/131 (32.8)		37/125 (29.6)		38/123 (30.9)	
Attainment of goal blood pressure						
Control	45/196 (23.0)	.73	48/186 (25.8)	.57	50/182 (27.5)	.23
Intervention A	29/143 (20.3)		38/138 (27.5)		45/136 (33.1)	
Intervention B	31/128 (24.2)		39/123 (31.7)		41/122 (33.6)	
Intervention C	33/131 (25.2)		40/125 (32.0)		48/123 (39.0)	

^a^ Intervention A was a letter; intervention B, a letter plus a financial incentive; and intervention C, a letter, financial incentive, and a telephone call encouraging patients to speak with their primary care clinicians.

^b^ χ^2^ Test of independence.

**Table 3.** Adjusted Results of Thiazide Prescribing and Blood Pressure Control at Index Visit, 6-Month Follow-up, and 12-Month Follow-up^a^

Outcome and Group^b^	Odds Ratio (95% CI)		
	Index Visit (n = 598)	Follow-up	
		6 mo (n = 572)	12 mo (n = 563)
Thiazide prescription			
Control	1 [Reference]	1 [Reference]	1 [Reference]
Intervention A	2.94 (1.56-5.54)^c^	1.58 (0.88-2.82)	1.24 (0.73-2.10)
Intervention B	3.24 (1.70-6.18)^c^	1.59 (0.87-2.89)	1.23 (0.71-2.14)
Intervention C	4.85 (2.60-9.05)^c^	2.50 (1.42-4.41)^d^	1.58 (0.93-2.67)
Attainment of goal blood pressure			
Control	NA	1 [Reference]	1 [Reference]
Intervention A	NA	1.04 (0.59-1.84)	1.33 (0.82-2.17)
Intervention B	NA	1.30 (0.73-2.30)	1.32 (0.80-2.20)
Intervention C	NA	1.05 (0.59-1.89)	1.73 (1.06-2.83)^e^

Abbreviation: NA, not applicable.

^a^ Intervention A was a letter; intervention B, a letter plus a financial incentive; and intervention C, a letter, financial incentive, and a telephone call encouraging patients to speak with their primary care clinicians.

^b^ Models adjusted for race/ethnicity and baseline blood pressure values (attainment of goal blood pressure).

^c^ *P* < .001.

^d^ *P* = .007.

^e^ *P* = .04.

The second primary outcome was attainment of goal BP. For reference, this outcome was similar across all groups at the index visit, with 20.3% of group A (29 of 143), 24.2% of group B (31 of 128), and 25.2% of group C (33 of 131) and 23.0% of controls (45 of 196) at their goal BP (*P* = .73). Because of the time gap between enrollment of those not at goal and the index visit, some patients previously not at goal were at their goal BP at the index measurement. For the control group, mean BP was 151.0/83.4 mm Hg at enrollment, 140.8/81.0 mm Hg at the index visit, and reduced to 137.7/79.3 mm Hg at the 6-month follow-up and 138.0/79.0 mm Hg at the 12-month follow-up; the mean decrease in systolic BP was 13.0 mm Hg. For the intervention groups, greater reductions in mean BP were seen from enrollment (148.1/83.8 mm Hg) to the index visit (139.8/80.6 mm Hg), the 6-month follow-up (135.6/79.4 mm Hg), and the 12-month follow-up (133.3/77.8 mm Hg); the mean decrease in systolic BP was 14.8 mm Hg (eFigure in Supplement 2). Unadjusted rates of attainment of BP goals increased for the control group and all 3 intervention groups, but differences between groups were not significant (Table 2). Adjusted odds ratios for the 6-month and 12-month follow-ups are presented in Table 3. Although there was no significant intervention effect at 6 months, there was a significant effect at 12 months for group C (adjusted odds ratio, 1.73; 95% CI, 1.06-2.83; *P* = .04). No significant clustering owing to physicians or clinics was observed; that is, the intercluster correlation coefficient values were zero.

The prespecified secondary process measure of interest was the rate of patient-initiated discussion about thiazides. The discussion rate was 44.1% for group A (63 of 143), 56.3% for group B (72 of 128), and 68.7% for group C (90 of 131) (χ^2^_8_ = 20.34; *P* = .004). Follow-up comparisons indicated that each intervention group rate was significantly different from one another, indicating that patient-initiated discussion about thiazides increased in a dose-response fashion as additional pieces were added. Thus, the added financial incentive and telephone call from a health educator had incremental effects on discussion rates.

## Discussion

This randomized clinical trial using a novel combination of 3 interventions based on the principles of patient activation resulted in high rates of evidence-based treatment of hypertension and modest achievement of BP goals compared with usual care. The first step in achieving these results was to activate the patients to engage their primary care clinicians. A customized letter alone resulted in almost half (44.1%) of participants engaging their primary care clinicians, with a financial incentive (56.3% of patients) and telephone call (68.7% of patients) adding incrementally to rates of patient activation. Application of this type of patient activation will be needed to augment other components of interventions to achieve the new lower BP goals.^12,26^

After activation of the patient, thiazide prescribing also increased in a dose-response fashion from 24.5% in group A (35 of 143) to 32.8% in group C (43 of 131) (*P* < .001) compared with controls (9.7%). Although thiazides are just one of several primary agents recommended by guidelines,^12,14^ this rate was considerably higher than reported in an observational study of usual care in which only 19.5% of patients received drug therapy despite having uncontrolled hypertension.^43^ This difference was sustained in our study at 6 months (*P* = .04), but the magnitude was reduced owing to increases in thiazide use in the control group (16.7% [31 of 186]) and reductions in each of the 3 intervention groups, likely because the drug was ineffective or discontinued owing to adverse effects. By 12 months, there was no significant difference, with the control group increasing to a 22.5% rate of thiazide prescribing (41 of 182) and intervention groups at rates of 25.7% in group A (35 of 136), 27.0% in group B (33 of 122), and 30.9% in group C (38 of 123). This finding suggests that intervention clinicians may have been “learning” to use thiazides through the patient activation letters that other patients were bringing to them. This type of “nudge” could be applied to other medication prescribing challenges.^36^ Additional analyses of the results will determine to what extent contamination occurred, but since most clinicians only had 2 to 3 patients enrolled during 18 months (the maximum enrolled was 15 for 1 clinician), the effect may have been minimal but worth exploring.

The second primary outcome, attainment of BP goal, is a quality metric and proxy for improved clinical outcomes. At the index visit, there was no difference in attainment of BP goal, which was expected as the intervention was intended to encourage prescribing at that visit. However, what was surprising was that, despite all participants not being at their goal BP on their last 2 clinic visits, almost one-fourth were now at their BP goal and thus would not need additional pharmacotherapy. Across all 3 intervention groups and the control group, BP control improved during the 12 months of the study, with more than 30% achieving their goal and group C having a significantly higher rate of control in adjusted analysis (odds ratio, 1.73; 95% CI, 1.06-2.83). The mean BP at enrollment was 148.1/83.8 mm Hg in the combined intervention groups and 151.0/83.4 mm Hg in the control group. By study end, both groups saw reductions in mean BP: to 133.3/77.8 mm Hg in the combined intervention groups (14.8–mm Hg reduction) and 138.0/79.0 mm Hg in the control group (13.0–mm Hg reduction). This 1.8–mm Hg systolic BP reduction is comparable to reductions achieved with audit and feedback (0.8 mm Hg) and clinician training (1.4 mm Hg) reported in a recent meta-analysis but not as great as the reduction achieved with team-based care with titration by nonphysicians (7.1 mm Hg).^26^ This reduction would have meaningful long-term clinical benefits, and for the 65% of participants whose BP was still not controlled, they were closer to their goal BP.

The finding of almost one-fourth of participants with uncontrolled BP achieving BP control at baseline and only one-third achieving BP control at 12 months speaks to the variable nature of BP readings and treatment effects and is expected in routine clinical care. The delay in improved BP control in the intervention group until 12 months may reflect the clinical inertia that exists when treating BP; this intervention may augment current practice and help overcome such inertia.

These results are noteworthy in that they were achieved with a relatively low-cost intervention, used the patient as the agent of change, and can be used for other conditions and treatments. First, the creation of the risk assessment letter was customized based on information extracted from the electronic medical record and provided a tangible action plan (ie, specific medication for hypertension) to address a quantifiable cardiovascular risk. This type of risk stratification is consistent with recommendations from the American College of Cardiology/American Heart Association High Blood Pressure Clinical Practice Guideline to identify patients with the greatest potential benefit, especially for those with a systolic BP of 130 to 139 mm Hg.^12^ These types of patient activation letters could be readily generated by health care systems for other conditions, such as hyperlipidemia and diabetes; for medication de-escalation for opioid use for chronic pain and proton pump inhibitor use for gastroesophageal reflux; and for prevention programs for vaccinations and cancer screening.

Despite the dose-response nature of the findings, the independent effect of the financial incentive and telephone call remains unclear. For financial incentives, there is a robust literature on how behavioral economics can incentivize health-related behaviors.^44,45,46^ Prior work describes patient^47^ and primary care clinician^48^ perspectives on use of financial incentives for treatment of hypertension. Although impressions were mostly positive, financial incentives to engage in routine interactions with primary care clinicians may create an expectation for recurrent incentives and may not be the best use of resources. The previsit telephone call by a health educator to remind patients to bring the letter and postcard and engage their primary care clinician was relatively easy, involved a health coach, and could be used in primary care to enhance other activities, such as medication reconciliation and preventive care. Future work should consider moderators of the patient activation effects, such as higher health literacy^49^ or a stronger predilection toward shared decision making that may respond more effectively to this type of intervention.^50,51^

### Limitations

This study has several limitations. First, BP recording used clinic sphygmomanometers with no standardized recording protocol. We used this pragmatic approach to better reflect real-world decision making for hypertension treatment. Second, the modest but feasible financial incentive was small ($20) with or without a refund of the patient’s copayment for medication ($48) and was provided only once. Prior qualitative work^47,48^ did not elicit a high level of enthusiasm by patients or clinicians for such incentives for routine medical care and may not be as effective as prize-based or larger incentives that have also been studied.^52,53^ Third, hypertension guidelines evolve over time, making some recommendations obsolete. Regardless, the study addresses the general issues of patient activation, achievement of a target BP, and evidence-based treatments. Fourth, study recruitment was completed in 2008, with a delay in submitting the final results until now. Prior published results described qualitative findings from patients^47^ and clinicians,^48^ but trial outcomes have not been published previously, to our knowledge. Fifth, the study was performed in Veterans Affairs facilities, which have a single medication formulary and copayments that might not be generalizable, and 98.3% of the participants were male.

## Conclusions

This novel and low-resource intervention used patient activation to encourage evidence-based therapy and achieve improved BP goals compared with usual care and can be applied to other clinical settings to overcome clinical inertia. The financial incentive, although effective, may have limited value in routine clinical care, whereas customized patient activation letters and telephone calls may be more practical elements. Engagement of patients may also have longer-term effects on willingness to engage primary care clinicians in discussions and may have the added benefit of encouraging evidence-based prescribing by clinicians for other patients. Future work should embrace computer applications and other telehealth modalities to activate patients through non–face-to-face interactions.

## Notes

Supplement 1. **Trial Protocol**

Supplement 2. **eAppendix 1.** Sample Letter #1 Groups A, B, C **eAppendix 2.** Sample Script for Nurse Educator Phone Call **eFigure.** Mean Systolic Blood Pressure by Group vs Control

Supplement 3. **Data Sharing Statement**

## References

[1] Nwankwo T, Yoon SS, Burt V, Gu Q Hypertension among adults in the United States: National Health and Nutrition Examination Survey, 2011-2012. NCHS Data Brief. 2013;(133):-. 24171916

[2] Berlowitz DR, Ash AS, Hickey EC, Inadequate management of blood pressure in a hypertensive population. N Engl J Med. 1998;339(27):1957-1963. doi:10.1056/NEJM199812313392701 9869666

[3] Borzecki AM, Wong AT, Hickey EC, Ash AS, Berlowitz DR Hypertension control: how well are we doing? Arch Intern Med. 2003;163(22):2705-2711. doi:10.1001/archinte.163.22.2705 14662624

[4] Colhoun HM, Dong W, Poulter NR Blood pressure screening, management and control in England: results from the health survey for England 1994. J Hypertens. 1998;16(6):747-752. doi:10.1097/00004872-199816060-00005 9663914

[5] Meissner I, Whisnant JP, Sheps SG, Detection and control of high blood pressure in the community: do we need a wake-up call? Hypertension. 1999;34(3):466-471. doi:10.1161/01.HYP.34.3.466 10489395

[6] Ong KL, Cheung BM, Man YB, Lau CP, Lam KS Prevalence, awareness, treatment, and control of hypertension among United States adults 1999-2004. Hypertension. 2007;49(1):69-75. doi:10.1161/01.HYP.0000252676.46043.18 17159087

[7] Garg JP, Elliott WJ, Folker A, Izhar M, Black HR; RUSH University Hypertension Service Resistant hypertension revisited: a comparison of two university-based cohorts. Am J Hypertens. 2005;18(5, pt 1):619-626. doi:10.1016/j.amjhyper.2004.11.021 15882544

[8] Milchak JL, Carter BL, Ardery G, Dawson JD, Harmston M, Franciscus CL; Joint National Committee on Prevention, Detection, Evaluation, and Treatment of High Blood Pressure Physician adherence to blood pressure guidelines and its effect on seniors. Pharmacotherapy. 2008;28(7):843-851. doi:10.1592/phco.28.7.843 18576899

[9] Wang TJ, Vasan RS Epidemiology of uncontrolled hypertension in the United States. Circulation. 2005;112(11):1651-1662. doi:10.1161/CIRCULATIONAHA.104.490599 16157784

[10] Gu Q, Burt VL, Dillon CF, Yoon S Trends in antihypertensive medication use and blood pressure control among United States adults with hypertension: the National Health and Nutrition Examination Survey, 2001 to 2010. Circulation. 2012;126(17):2105-2114. doi:10.1161/CIRCULATIONAHA.112.096156 23091084

[11] Ho PM, Masoudi FA, Peterson ED, Cardiology management improves secondary prevention measures among patients with coronary artery disease. J Am Coll Cardiol. 2004;43(9):1517-1523. doi:10.1016/j.jacc.2003.12.037 15120805

[12] Whelton PK, Carey RM, Aronow WS, 2017 ACC/AHA/AAPA/ABC/ACPM/AGS/APhA/ASH/ASPC/NMA/PCNA guideline for the prevention, detection, evaluation, and management of high blood pressure in adults: a report of the American College of Cardiology/American Heart Association Task Force on Clinical Practice Guidelines [published correction appears in *J Am Coll Cardiol*. 2018;71 (19):2275-2279]. J Am Coll Cardiol. 2018;71(19):e127-e248. doi:10.1016/j.jacc.2017.11.006 29146535

[13] Spranger CB, Ries AJ, Berge CA, Radford NB, Victor RG Identifying gaps between guidelines and clinical practice in the evaluation and treatment of patients with hypertension. Am J Med. 2004;117(1):14-18. doi:10.1016/j.amjmed.2004.01.024 15210383

[14] James PA, Oparil S, Carter BL, 2014 Evidence-based guideline for the management of high blood pressure in adults: report from the panel members appointed to the Eighth Joint National Committee (JNC 8). JAMA. 2014;311(5):507-520. doi:10.1001/jama.2013.284427 24352797

[15] Appel LJ The verdict from ALLHAT—thiazide diuretics are the preferred initial therapy for hypertension. JAMA. 2002;288(23):3039-3042. doi:10.1001/jama.288.23.3039 12479770

[16] Chobanian AV, Bakris GL, Black HR, ; National Heart, Lung, and Blood Institute Joint National Committee on Prevention, Detection, Evaluation, and Treatment of High Blood Pressure; National High Blood Pressure Education Program Coordinating Committee The Seventh Report of the Joint National Committee on Prevention, Detection, Evaluation, and Treatment of High Blood Pressure: the JNC 7 report. JAMA. 2003;289(19):2560-2572. doi:10.1001/jama.289.19.2560 12748199

[17] Simon SR, Majumdar SR, Prosser LA, Group versus individual academic detailing to improve the use of antihypertensive medications in primary care: a cluster-randomized controlled trial. Am J Med. 2005;118(5):521-528. doi:10.1016/j.amjmed.2004.12.023 15866255

[18] Chalmers J Implementation of guidelines for management of hypertension. Clin Exp Hypertens. 1999;21(5-6):647-657. doi:10.3109/10641969909060996 10423089

[19] Swales JD Current clinical practice in hypertension: the EISBERG (Evaluation and Interventions for Systolic Blood Pressure Elevation–Regional and Global) project. Am Heart J. 1999;138(3, pt 2):231-237. doi:10.1016/S0002-8703(99)70315-7 10467218

[20] Artinian NT, Washington OG, Templin TN Effects of home telemonitoring and community-based monitoring on blood pressure control in urban African Americans: a pilot study. Heart Lung. 2001;30(3):191-199. doi:10.1067/mhl.2001.112684 11343005

[21] Murray MD, Harris LE, Overhage JM, Failure of computerized treatment suggestions to improve health outcomes of outpatients with uncomplicated hypertension: results of a randomized controlled trial. Pharmacotherapy. 2004;24(3):324-337. doi:10.1592/phco.24.4.324.33173 15040645

[22] Borenstein JE, Graber G, Saltiel E, Physician-pharmacist comanagement of hypertension: a randomized, comparative trial. Pharmacotherapy. 2003;23(2):209-216. doi:10.1592/phco.23.2.209.32096 12587810

[23] Walsh JM, McDonald KM, Shojania KG, Quality improvement strategies for hypertension management: a systematic review. Med Care. 2006;44(7):646-657. doi:10.1097/01.mlr.0000220260.30768.32 16799359

[24] Walsh JM, Sundaram V, McDonald K, Owens DK, Goldstein MK Implementing effective hypertension quality improvement strategies: barriers and potential solutions. J Clin Hypertens (Greenwich). 2008;10(4):311-316. doi:10.1111/j.1751-7176.2008.07425.x 18401229 PMC8110041

[25] Carter BL, Rogers M, Daly J, Zheng S, James PA The potency of team-based care interventions for hypertension: a meta-analysis. Arch Intern Med. 2009;169(19):1748-1755. doi:10.1001/archinternmed.2009.316 19858431 PMC2882164

[26] Mills KT, Obst KM, Shen W, Comparative effectiveness of implementation strategies for blood pressure control in hypertensive patients: a systematic review and meta-analysis. Ann Intern Med. 2018;168(2):110-120. doi:10.7326/M17-1805 29277852 PMC5788021

[27] Watkins CJ, Papacosta AO, Chinn S, Martin J A randomized controlled trial of an information booklet for hypertensive patients in general practice. J R Coll Gen Pract. 1987;37(305):548-550. 3503941 PMC1711180

[28] Hunt JS, Siemienczuk J, Touchette D, Payne N Impact of educational mailing on the blood pressure of primary care patients with mild hypertension. J Gen Intern Med. 2004;19(9):925-930. doi:10.1111/j.1525-1497.2004.40046.x 15333056 PMC1492526

[29] Mintzes B, Barer ML, Kravitz RL, How does direct-to-consumer advertising (DTCA) affect prescribing? a survey in primary care environments with and without legal DTCA. CMAJ. 2003;169(5):405-412. 12952801 PMC183290

[30] Kravitz RL Direct-to-consumer advertising of prescription drugs: implications for the patient-physician relationship. JAMA. 2000;284(17):2244. doi:10.1001/jama.284.17.2244-JMS1101-5-1 11056603

[31] Kravitz RL, Epstein RM, Feldman MD, Influence of patients’ requests for direct-to-consumer advertised antidepressants: a randomized controlled trial. JAMA. 2005;293(16):1995-2002. doi:10.1001/jama.293.16.1995 15855433 PMC3155410

[32] Hibbard JH, Stockard J, Mahoney ER, Tusler M Development of the Patient Activation Measure (PAM): conceptualizing and measuring activation in patients and consumers. Health Serv Res. 2004;39(4, pt 1):1005-1026. doi:10.1111/j.1475-6773.2004.00269.x 15230939 PMC1361049

[33] Greene J, Hibbard JH Why does patient activation matter? an examination of the relationships between patient activation and health-related outcomes. J Gen Intern Med. 2012;27(5):520-526. doi:10.1007/s11606-011-1931-2 22127797 PMC3326094

[34] Singer S, Shortell SM Implementing accountable care organizations: ten potential mistakes and how to learn from them. JAMA. 2011;306(7):758-759. doi:10.1001/jama.2011.1180 21828308

[35] Hibbard JH, Greene J, Overton V Patients with lower activation associated with higher costs; delivery systems should know their patients’ ‘scores’. Health Aff (Millwood). 2013;32(2):216-222. doi:10.1377/hlthaff.2012.1064 23381513

[36] Avorn J The psychology of clinical decision making—implications for medication use. N Engl J Med. 2018;378(8):689-691. doi:10.1056/NEJMp1714987 29466158

[37] Pfeiffer E A short portable mental status questionnaire for the assessment of organic brain deficit in elderly patients. J Am Geriatr Soc. 1975;23(10):433-441. doi:10.1111/j.1532-5415.1975.tb00927.x 1159263

[38] CONSORT 2010. CONSORT website. http://www.consort-statement.org/consort-2010. Accessed September 5, 2018.

[39] Wilson PW, D’Agostino RB, Levy D, Belanger AM, Silbershatz H, Kannel WB Prediction of coronary heart disease using risk factor categories. Circulation. 1998;97(18):1837-1847. doi:10.1161/01.CIR.97.18.1837 9603539

[40] National Cholesterol Education Program (NCEP) Expert Panel on Detection, Evaluation, and Treatment of High Blood Cholesterol in Adults (Adult Treatment Panel III) Third Report of the National Cholesterol Education Program (NCEP) Expert Panel on Detection, Evaluation, and Treatment of High Blood Cholesterol in Adults (Adult Treatment Panel III) final report. Circulation. 2002;106(25):3143-3421. doi:10.1161/circ.106.25.3143 12485966

[41] Kaboli PJ, McClimon BJ, Hoth AB, Barnett MJ Assessing the accuracy of computerized medication histories. Am J Manag Care. 2004;10(11, pt 2):872-877. 15609741

[42] Hedeker D, Gibbons RD, Flay BR Random-effects regression models for clustered data with an example from smoking prevention research. J Consult Clin Psychol. 1994;62(4):757-765. doi:10.1037/0022-006X.62.4.757 7962879

[43] Khanna RR, Victor RG, Bibbins-Domingo K, Shapiro MF, Pletcher MJ Missed opportunities for treatment of uncontrolled hypertension at physician office visits in the United States, 2005 through 2009. Arch Intern Med. 2012;172(17):1344-1345. doi:10.1001/archinternmed.2012.2749 22869238

[44] Halpern SD, French B, Small DS, Randomized trial of four financial-incentive programs for smoking cessation. N Engl J Med. 2015;372(22):2108-2117. doi:10.1056/NEJMoa1414293 25970009 PMC4471993

[45] Volpp KG, John LK, Troxel AB, Norton L, Fassbender J, Loewenstein G Financial incentive-based approaches for weight loss: a randomized trial. JAMA. 2008;300(22):2631-2637. doi:10.1001/jama.2008.804 19066383 PMC3583583

[46] Volpp KG, Loewenstein G, Troxel AB, A test of financial incentives to improve warfarin adherence. BMC Health Serv Res. 2008;8:272. doi:10.1186/1472-6963-8-272 19102784 PMC2635367

[47] Pilling SA, Williams MB, Brackett RH, Part I, patient perspective: activating patients to engage their providers in the use of evidence-based medicine: a qualitative evaluation of the VA Project to Implement Diuretics (VAPID). Implement Sci. 2010;5:23. doi:10.1186/1748-5908-5-23 20298563 PMC2850871

[48] Buzza CD, Williams MB, Vander Weg MW, Christensen AJ, Kaboli PJ, Reisinger HS Part II, provider perspectives: should patients be activated to request evidence-based medicine? a qualitative study of the VA Project to Implement Diuretics (VAPID). Implement Sci. 2010;5:24. doi:10.1186/1748-5908-5-24 20298564 PMC2856519

[49] Mosher HJ, Lund BC, Kripalani S, Kaboli PJ Association of health literacy with medication knowledge, adherence, and adverse drug events among elderly veterans. J Health Commun. 2012;17(suppl 3):241-251. doi:10.1080/10810730.2012.712611 23030573

[50] Cvengros JA, Christensen AJ, Cunningham C, Hillis SL, Kaboli PJ Patient preference for and reports of provider behavior: impact of symmetry on patient outcomes. Health Psychol. 2009;28(6):660-667. doi:10.1037/a0016087 19916633

[51] Kaboli PJ, Baldwin AS, Henderson MS, Ishani A, Cvengros JA, Christensen AJ Measuring preferred role orientations for patients and providers in Veterans Administration and university general medicine clinics. Patient. 2009;2(1):33-38. doi:10.2165/01312067-200902010-00004 22273057

[52] Halpern SD, Kohn R, Dornbrand-Lo A, Metkus T, Asch DA, Volpp KG Lottery-based versus fixed incentives to increase clinicians’ response to surveys. Health Serv Res. 2011;46(5):1663-1674. doi:10.1111/j.1475-6773.2011.01264.x 21492159 PMC3207198

[53] Volpp KG, Asch DA, Galvin R, Loewenstein G Redesigning employee health incentives—lessons from behavioral economics. N Engl J Med. 2011;365(5):388-390. doi:10.1056/NEJMp1105966 21812669 PMC3696722

