# Supplementary material for: Efficacy of Patient Activation Interventions With or Without Financial Incentives to Promote Prescribing of Thiazides and Hypertension Control: A Randomized Clinical Trial
Source: JAMA Netw Open. 2018 Dec 14;1(8):e185017. doi: 10.1001/jamanetworkopen.2018.5017 (PMC6324341; doi:10.1001/jamanetworkopen.2018.5017)
Supplement: Supplement 1. — Trial Protocol [file jamanetwopen-1-e185017-s001.pdf]

(D.) METHODS: VA PROJECT to IMPLEMENT DIURETICS (VAPID)

D.1 Overview

This 36-month randomized controlled trial will be conducted at 2 VA medical centers (VAMCs) and 4 affiliated community-based outpatient clinics (CBOCs) in VISN 23. The study will enroll 900 randomly selected primary care patients with hypertension not currently taking a thiazide and either:

1) **Not at** an appropriate treatment goal: systolic blood pressure $\geq$ 140mmHg (130mmHg for diabetic patients) or diastolic blood pressure $\geq$ 90mmHg (80mmHg for diabetics); or
2) **At** appropriate blood pressure goal, but currently taking a calcium channel blocker (CCB).

The study will employ a multi-step randomization protocol involving physicians and patients. (**Figure 2**) ***First***, primary care providers at both the VAMCs and CBOCs will be randomized to intervention or control groups in an 8:1 manner such that 160 providers will be designated intervention providers and 20 will be designated control providers. ***Second***, patients of control and intervention providers will be stratified according to whether or not they are at an appropriate treatment goal. ***Third***, 50 patients not at treatment goal and 50 patients at treatment goal (but on a CCB) of **control physicians** will be randomly identified and will serve as a control group (*Control Group 1*). ***Fourth***, 400 patients not at treatment goal and 400 patients at goal (but on a CCB) of **intervention physicians** will be randomized to a control group (C*ontrol Group 2*) or one of three intervention arms that incrementally build on each other:

Within each of the control and intervention groups, roughly one-half of patients will not be at treatment goal and one-half will be at treatment goal, but will be taking a CCB. In addition, the patient recruitment process will seek to randomize equivalent numbers of patients from VAMCs and CBOCs in Minnesota and Iowa. Patients randomized to control groups will receive usual care with their primary care provider and follow-up care as scheduled. Patients can only be enrolled in the study once during the study period.

Patients will be enrolled into the study over an 18-month period (months 4-21) and will be followed for 12 months after enrollment. Study data will be obtained from VA administrative data files, patient medical records, and patient interviews. ***Primary endpoints*** include: 1) the *percent of patients taking thiazide diuretics* at their index visit and at 6 and 12 months; 2) the *percent of patients at blood pressure treatment goal* at 6 and 12 months; and 3) *mean blood* pressure at 6 and 12 months.

Figure 2. Randomization Protocol

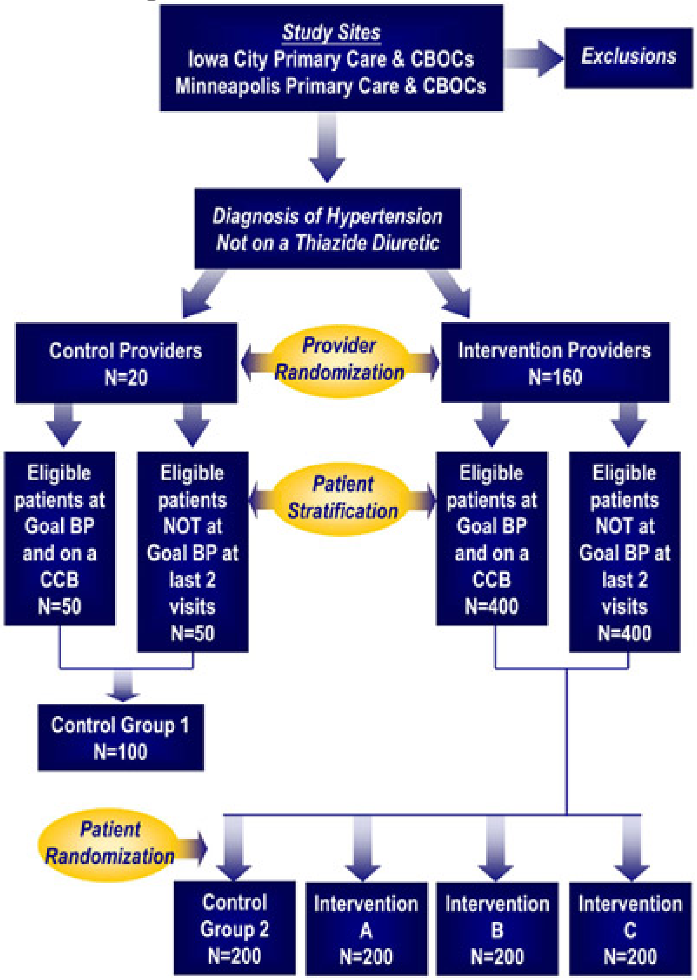

D.5. Intervention Description

The proposed patient-activation intervention is designed to increase the use of thiazide diuretics and improve the quality of hypertension management. All arms of the intervention incorporate a tailored patient education mailing to activate patients to initiate a discussion of the use of thiazides with their primary care provider. The study will also test the incremental utility of a financial incentive and a follow-up health-educator telephone call. After patient informed consent and randomization, all patients will complete baseline data collection forms over the telephone, then instructed that they will be contacted at a later time as part of the study. Control patients will not be contacted again until 6 months after their index clinic visit. Intervention patients will be randomly assigned to one of three intervention groups.

D.5.i. Intervention Group A will receive a customized patient educational letter providing cardiovascular risk assessment based on the Framingham Heart Study mailed to them 3 weeks prior to their scheduled clinic appointment. Draft versions of the letters and post cards are attached, (Appendix 1) with the final format and design being determined during the pilot phase. (Months 1-3) Information will be customized for the patient's current treatment regimen and blood pressure. For patients *not at their blood pressure goal*, the initial letter will: i) address prior blood pressures, ii) identify their blood pressure goal, iii) quantify the cardiovascular risk reduction with achieving the goal, and iv) provide suggestions for reaching that goal, namely the addition of a thiazide diuretic. It will specifically request the patient initiate a discussion with their provider about blood pressure treatment with a thiazide. This letter will be followed up in 1 week with another letter reminding them to discuss this with their provider, including a post card for them to send back after their appointment documenting whether they discussed their hypertension and a place for their provider to sign it confirming the discussion took place. The letters will be worded differently for patients at their blood pressure goal and on a CCB, suggesting they be switched to a thiazide diuretic.

D.5.ii. Intervention Group B will receive the same initial letter, but their follow-up letter will describe the financial incentive of a one-time $20 rebate to initiate a discussion with their provider. Patients will be mailed a rebate check upon receipt of their signed post-card. For patients with a co-pay, the letter will also tell them their first 6 months of a thiazide will be free through a $42 rebate that will be mailed to them upon receipt of the post card and confirmation that a thiazide was prescribed.

D.5.iii Intervention Group C will receive the same initial letter and a similar follow-up letter, but it will include an additional line stating they will receive a telephone call to answer any questions before their next clinic visit. Previous research involving patients being treated for HTN have shown that phone call reminders are effective in increasing compliance with treatment recommendations[42] and a recent intervention of a mailed video to encourage colon cancer screening was ineffective and the investigators suggested that their intervention may have been more effective if patients were called prior to their clinic visit and reminded to ask their providers about colon cancer screening.[85] A nurse-educator will attempt to call the patient 3 days prior to their clinic visit, allowing for repeated calls over the next 2 days if the patient is not available. She will ask the patient if they received the information about their blood pressure, briefly review the information and ask if they have any questions about it. She will then ask them how they feel about bringing up the possibility of changing one of their medications with their providers. If they have any reservations, she will offer encouragement and support.

Follow-up telephone calls for 6 and 12-month data collection will be performed using a method proven successful in the EPOC study. All data collection forms will be mailed to the patient 1-week prior to a telephone call. Each form will be labeled with colored letter stickers beginning with the letter "A". During the phone interview, the research assistant will ask the subject to begin with form "A" and will ask the questions over the phone. In our past experience, this was more successful than depending on forms to be mailed back, or having subjects answer questions without the forms in front of them.

Table 2) Summary of Data Elements and Sources and Timing of Data Collection

Data Element	Source of Information	Base line	Index visit	6 Mos	12 Mos
Primary Endpoints					
• Thiazide diuretic use	VA Medical record		X	X	X
• Attainment of Target Blood Pressure	VA Medical record	X	X	X	X
• Mean Blood Pressure Change	VA Medical record	X	X	X	X
Secondary Endpoints					
• Discussion of Hypertension Treatment with Provider	Mail-back card<br>Patient interview		X		
• Hypertension Medication Number and Costs	VA pharmacy database<br>Patient interview	X		X	X
• Adverse Drug Events Related to Anti-hypertensive Medication	Patient interview<br>Chart review			X	
• Hypertension Medication Compliance	Morisky scale, VA database			X	
• VA Clinic and Urgent Care Visits	VA database			X	X
• VA Hospitalizations	VA database			X	X
• Non-VA Clinic & Emergency Room Visits	Patient interview			X	X
• Non-VA Hospitalizations	Patient interview			X	X
• Laboratory Testing	VA database			X	X
• Knowledge of Blood Pressure Medications	Patient interview	X		X	X
• Cost of Intervention	VA personnel/supply costs				X
Control Variables					
• Demographics / socioeconomic status	Patient interview, VA data	X			
• Comorbidity	Medical records	X			
• Non-VA primary care co-management	Patient interview	X		X	X
• Patient Provider Orientation Scale	Patient and Provider Survey	X			
• Pfeiffer Mental Status Questionnaire	Patient interview	X			

D.7. Data Analysis

D.7.i. Data Inspection and baseline comparison of treatment groups: Data elements will be examined using univariate statistics and graphical techniques. Ordinal and continuous measures will be described using medians, means, standard deviations, and 95% confidence intervals; data distributions will be examined for violations of normality. Dichotomous and nominal variables will be summarized as proportions. Intervention and control sub-groups will be compared at baseline in terms of socio-demographic characteristics and other variables (e.g. comorbidity, non-VA co-management, PPOS scores, mental status) using ANOVA, Kruskal-Wallis, or chi-squared tests as appropriate. Variables showing a significant difference between the treatment groups will be included as covariates in adjusted analyses.

D.7.ii. Statistical Analyses: Hypothesis testing will compare endpoints in intervention and control groups at baseline and 6 and 12-month follow-up. Analyses will use logistic regression for dichotomous responses and linear regression for continuous endpoints. All models will incorporate random physician effects to account for within-physician clustering. Study sites will be included as fixed effects in all models. Models specific to each of the study hypotheses are described in the following sections.

Hypothesis 1a: *The use of thiazide diuretics will be higher in patients receiving individualized risk information, compared to control patients after their index visit and 6 and 12-month follow-up.*

Hypothesis 1b: The use of thiazides will be higher in intervention patients receiving an additional financial incentive and/or a health educator telephone call compared to intervention patients receiving the risk information alone after their index visit and 6 and 12 months.

For each time point (index, 6, 12 months), let Y_{ijkl} be the indicator of thiazide use for patient *i* of physician *j* in treatment group *k*, where *k* = 1-5. If the patient is at goal blood pressure at baseline, then *l* = 1; otherwise *l* = 2. We assume $Y_{ilkj} \sim$ binomial $(1, p_{ijkl})$. Hypotheses 1a and 1b will be tested using the model

$$\log \frac{p_{ijkl}}{1 - p_{ijkl}} = \mu + \pi_j + (\text{treatment})_k + (\text{BPgoal})_l + (\text{treatment} \times \text{BPgoal})_{jl} + \mathbf{X\beta}$$

where π_j are provider random effects with independent $N(0, \sigma^2)$ distributions, $(\text{treatment})_j$ and $(\text{BPgoal})_l$ are dummy indicators for treatment and goal blood pressure baseline status, $(\text{treatment X BPgoal})_{jl}$ is the interaction term, and $\mathbf{X\beta}$ are covariate effects at either the patient or physician level; these include the study sites as dummy variables and baseline covariates that were significantly different across study groups. Using appropriate contrasts, we will test separately after index visit, 6, and 12 months if there is a significant difference between the log odds of thiazide use for the pure controls versus the average of the 3 interventions. A significant difference will be followed by pairwise comparisons between the pure control and each of the 3 interventions for *Hypothesis 1a,* and among the 3 interventions for *Hypothesis 1b*.

Hypothesis 2a: In patients not at goal blood pressure before the intervention, mean blood pressure will be lower in the intervention groups compared to control patients at 6 and 12 months.

Hypothesis 2b: In patients at blood pressure goal, but on a guideline discordant medication (CCBs), mean blood pressure will be similar between control and intervention groups at 6 and 12 months.

Let Y_{ijk} denote blood pressure for patient *i* seeing physician *j* in treatment group *k*. These hypotheses will be tested separately at 6 and 12 months using the model

$$Y_{ijk} = \mu + \pi_j + (\text{treatment})_k + \mathbf{X\beta} + \varepsilon_{ijk}$$

where π_j and $\mathbf{X\beta}$ are defined as before. We restrict the analysis to those patients not at goal blood pressure for *Hypothesis 2a*, and restrict the analysis to those patients at goal blood pressure and on guideline discordant medication for *Hypothesis 2b*. *Hypothesis 2a* will be tested by comparing the average of the 3 intervention group means with the pure control group. A significance difference will be followed by pairwise comparisons. *Hypothesis 2b* will be tested similarly.

Hypothesis 3a: Intervention patients who expect to play active roles in the clinical encounter will be more likely to discuss the patient education material with their provider, be started on a thiazide, and have lower post-intervention blood pressure than patients who expect to play a more passive role.

Hypothesis 3b: The effect of the patient activation intervention on thiazide use and blood pressure will be highest among patients whose providers believe that patients should be actively involved in treatment decision-making (a more "patient centered" role orientation) relative to providers who believe patients should adopt a more passive role (a more "provider centered" orientation).

If thiazide use or patient-provider discussion is the outcome, analyses for Hypothesis 3a will be restricted to the 3 interventions; if blood pressure is the endpoint, analyses will additionally be restricted to patients not at blood pressure goal at baseline. The 3 intervention groups will be combined to increase power. Separate analyses will be performed at 6 and 12 months. For analyses of thiazide use and patient-provider discussion, the model is:

$$\log \frac{p_{ijkl}}{1 - p_{ijkl}} = \mu + \pi_j + \beta_1 (\text{PPOS})_{ij} + (\text{intervention})_k + (\text{BPgoal})_l + \mathbf{X\beta}$$

Of interest is the test for β_1, with exp (β_1) being the odds ratio for each 1 unit increment in the Patient-Provider Orientation Scale (PPOS). We will also examine the joint influence by including both the

physician-level and patient-level PPOS scores and their interaction in a model. Similar linear models will be constructed for the blood pressure outcome.

D.7.iii. Sample size: For our sample size estimates we assume that after attrition we are left with 740 subjects in the intervention arm (185 in each intervention group, 148 providers) and 90 subjects in the control arm for 18 providers. *All power estimates are somewhat conservative since baseline covariates will be included in the models, which will reduce the standard errors.*

Hypothesis 1a and 1b: Sample sizes were selected to provide sufficient (≥ 80%) power to detect an 18% absolute difference in thiazide use between the pure control group and a single treatment group, and a 15% difference between any 2 intervention groups if the within-provider correlation is as high as .10, a conservative estimate. Power computations are summarized in **Table 1**.

Table 1. Summary of Study Power for Hypotheses 1a & 1b.

Hypothesis	*Effect size (% difference)*	*Intra-provider correlation*	*Power*
1a:Control Group 1	18	.05	0.85
vs. Intervention	18	.10	0.81
	20	.05	0.92
	20	.10	0.90
1b:Intervention vs.	15	≥ 0	0.80
Intervention	20	≥ 0	0.97

Hypothesis 2a and 2b: The sample sizes will be half as large (i.e., 45 control subjects, 92 intervention subjects). We have 80% power to detect a .54 standard deviation difference in blood pressure between the pure control and a single intervention group. This estimate is computed similar to that for Aim 1 with intra-provider correlation = .10, except that the binomial variance formula is replaced by the variance and that we assume the 45 control subjects are distributed in 9 clusters of 2 and 9 clusters of 3, and that the 92 intervention subjects each have a different provider.

D.10. Assurance of Patient Safety/Human Subjects

Our study involves minimal risk to patients and providers, however informed consent will be obtained. For the 20 providers in *Control Group 1*, no identifying data will be collected nor will we ask them to complete any surveys, so we will not require informed consent. For the 160 providers in the intervention arms and *Control Group 2*, we will provide informed consent at the time we ask them to complete the Patient-Provider Orientation Scale and notify them that they will have patients in the study.

The intent of the intervention is to activate patients to engage their providers to initiate changes in blood pressure medications. All medications are associated with actual and potential adverse drug events. Because these changes are made within the context of care with their primary care provider, we do not feel we are placing the patients at increased risk. The primary provider will have final say in all medication changes and the study itself does not initiate any therapy. Thiazide diuretics have a long history of safety and in the ALLHAT trial, chlorthalidone was better tolerated than lisinopril, but did have higher rates of hypokalemia and new diabetes.[9] We will work closely with the Institutional Review Boards in Iowa City and Minneapolis to develop an informed consent document that addresses all reasonable potential risk to subjects. There is minimal risk associated with data confidentiality, however all subjects will be assigned unique subject numbers and the study database will be kept on a secure server at the Iowa City VAMC. Encrypted data will be sent from Minneapolis to Iowa City by CD-ROM.
