# Supplementary material for: Efficacy of Patient Activation Interventions With or Without Financial Incentives to Promote Prescribing of Thiazides and Hypertension Control: A Randomized Clinical Trial
Source: JAMA Netw Open. 2018 Dec 14;1(8):e185017. doi: 10.1001/jamanetworkopen.2018.5017 (PMC6324341; doi:10.1001/jamanetworkopen.2018.5017)
Supplement: Supplement 2. — eAppendix 1. Sample Letter #1 Groups A, B, C eAppendix 2. Sample Script for Nurse Educator Phone Call eFigure. Mean Systolic Blood Pressure by Group vs Control [file jamanetwopen-1-e185017-s002.pdf]

Supplementary Online Content

Kaboli PJ, Howren MB, Ishani A, Carter B, Christensen AJ, Vander Weg MW. Efficacy of patient activation interventions with or without financial incentives to promote prescribing of thiazides and hypertension control: a randomized clinical trial. *JAMA Netw Open.* 2018;1(8):e185017. doi:10.1001/jamanetworkopen.2018.5017

eAppendix 1. Sample Letter #1 Groups A, B, C
eAppendix 2. Sample Script for Nurse Educator Phone Call
eFigure. Mean Systolic Blood Pressure by Group vs Control

This supplementary material has been provided by the authors to give readers additional information about their work.

© 2018 Kaboli PJ et al. *JAMA Network Open.*

DEPARTMENT OF VETERANS AFFAIRS
Medical Center
Iowa City, Iowa 52246-2208

eAppendix 1. Sample Letter #1 Groups A, B, C***
October 1, 2005

Dear Mr. Johnson,

The Iowa City VA Medical Center wants you to have the best possible health care and make sure your blood pressure is controlled. Your last blood pressures recorded in the clinic were **150/56** and **150/68**, which are **too high**. Your blood pressure should be kept below **140/90**. High blood pressure can cause heart attacks and strokes.

If you could lower your blood pressure to **120/80**, your 10-year risk of having a heart attack or stroke would be reduced by **35%.** That is a pretty big improvement!

How can we work together to reduce your blood pressure?

- Lifestyle changes like exercising and limiting your salt intake can lower your blood pressure. Your doctor can tell you more about things you can do.

- We can also adjust your blood pressure medication. You are currently taking:
  - **Amlodipine** **10 mg, 1 tablet a day**
  - **Atenolol** **50 mg, 1 tablet a day**

- According to blood pressure treatment guidelines set by the VA, the best medication for the treatment of high blood pressure in most patients is a low-dose of a **diuretic**, also called a water pill. **Diuretics** are safe and help reduce your chances of having a heart attack or stroke.

Possible options for you would be to:

- Switch to a combination pill that has Atenolol plus a diuretic (**Atenolol 50mg/chlorthalidone 25mg**)

  or
- Add a tablet of **chlorthalidone 25mg** or **HCTZ 25mg**.

Take this letter with you to your next doctor's appointment on **October 15th**. Ask if a **diuretic** is right for you to get your blood pressure down to where it should be. We are committed to working with you to provide you, the best possible health care.

The Iowa City VA Medical Center

© 2018 Kaboli PJ et al. *JAMA Network Open.*

eAppendix 2. Sample Script for Nurse Educator Phone Call***

"Hello, may I speak with Mr. Johnson please? This is Jane Morse, a nurse with the VA clinic where you see Dr. Smith. How are you today?"

"The reason I am calling is to follow-up on a letter we sent you last week about your blood pressure. In this letter, we told you your last two clinic blood pressures were too high and wanted to work with you to get your blood pressure down. Do you remember receiving this letter?" (*Script will need customization based upon patient's responses. Different script will be developed for patients on a CCB and at their blood pressure goal.)*

"In this letter, we suggested that a diuretic, or a water pill, might be right for you to help get your blood pressure down. We know that a low-dose of a diuretic is the best possible medication to reduce your risk of strokes and heart attacks from high blood pressure. We would like you to ask Dr. Smith about the possibility of starting a diuretic for your blood pressure. Do you feel comfortable asking him about this?" (*Script will need customization based upon patient's responses.)*

"If you don't mind, please take the reminder post card with you to your visit and have Dr. Smith sign it when you discuss your blood pressure and send it back to us. We will send you $20 just for having the discussion with Dr. Smith." (*If patient has a co-pay, will need to remind them we will send them a rebate for their first six months of a diuretic*.)

"Do you have any questions for me?"

"Thank you for your time. The VA is committed to making sure you have the best possible care and we hope this helps in the management of your high blood pressure. Have a nice day."

Note: Please record any comments or suggestions the patient has about the phone call or questions they may have had.

© 2018 Kaboli PJ et al. *JAMA Network Open.*

eFigure. Mean Systolic Blood Pressure by Group vs Control

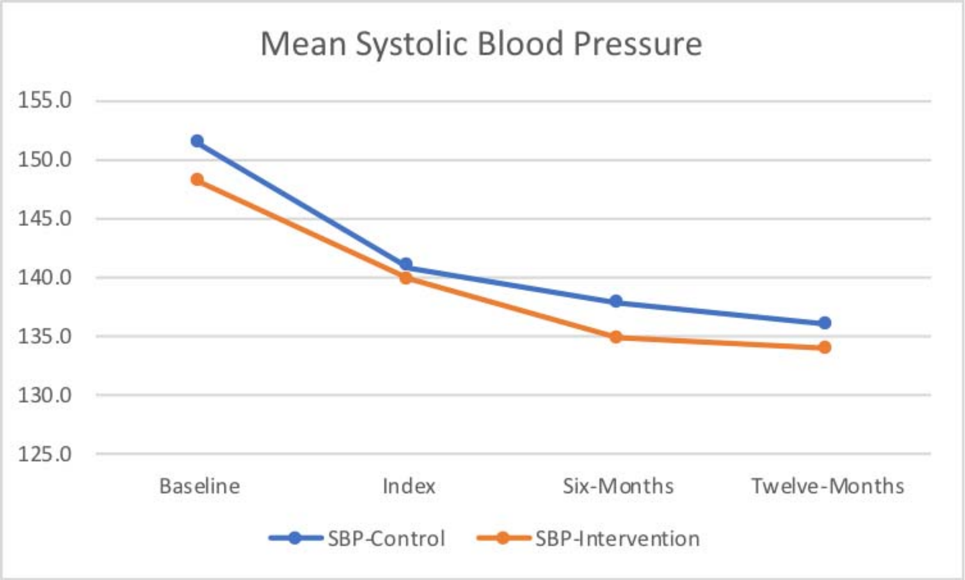

Note: Intervention mean collapsed across intervention groups A, B, C.

© 2018 Kaboli PJ et al. *JAMA Network Open.*
