# Supplementary material for: Efficacy of Patient Activation Interventions With or Without Financial Incentives to Promote Prescribing of Thiazides and Hypertension Control: A Randomized Clinical Trial
Source: JAMA Netw Open. 2018 Dec 14;1(8):e185017. doi: 10.1001/jamanetworkopen.2018.5017 (PMC6324341; doi:10.1001/jamanetworkopen.2018.5017)
Supplement: Supplement 3. — Data Sharing Statement [file jamanetwopen-1-e185017-s003.pdf]

Data Sharing Statement

Kaboli. Efficacy of Patient Activation Interventions With or Without Financial Incentives to Promote Prescribing of Thiazides and Hypertension Control. *JAMA Netw Open*. Published December 14, 2018. 10.1001/jamanetworkopen.2018.5017

Data
Data available: No

Additional Information
Explanation for why data not available: We are not allowed to release VA data to others without local approvals. All VA data must remain within the VA firewall.
